# Sex-Specific Associations of Blood-Based Nutrient Profiling With Body Composition in the Elderly

**DOI:** 10.3389/fphys.2018.01935

**Published:** 2019-01-24

**Authors:** Tobias Konz, Aurelia Santoro, Laurence Goulet, Alberto Bazzocchi, Giuseppe Battista, Claudio Nicoletti, Fawzi Kadi, Rita Ostan, Michael Goy, Caroline Monnard, François-Pierre Martin, Jerome N. Feige, Claudio Franceschi, Serge Rezzi

**Affiliations:** ^1^Nestlé Research, Vers-Chez-Les-Blanc, Lausanne, Switzerland; ^2^Department of Experimental, Diagnostic and Specialty Medicine, Alma Mater Studiorum, University of Bologna, Bologna, Italy; ^3^C.I.G. Interdepartmental Centre “L. Galvani”, Alma Mater Studiorum, University of Bologna, Bologna, Italy; ^4^Diagnostic and Interventional Radiology, IRCCS Istituto Ortopedico Rizzoli, Bologna, Italy; ^5^Department of Experimental and Clinical Medicine, Section of Anatomy, University of Florence, Florence, Italy; ^6^Gut Health Institute Strategic Programme, Quadram Institute Bioscience, Norwich, United Kingdom; ^7^School of Health and Medical Sciences, Örebro University, Örebro, Sweden; ^8^Nestlé Research, EPFL Innovation Park, Lausanne, Switzerland; ^9^Institute of Neurological Sciences (IRCCS), Bologna, Italy

**Keywords:** nutrient profiling, nutritional status, body composition, elderly, minerals, trace elements, fatty acids, vitamins

## Abstract

The intake of adequate amounts and types of nutrients is key for sustaining health and a good quality of life, particularly in the elderly population. There is considerable evidence suggesting that physiological changes related to age and sex modify nutritional needs, and this may be related to age-associated changes in body composition (BC), specifically in lean and fat body mass. However, there is a clear lack of understanding about the association of nutrients in blood and BC parameters in the elderly. This study investigated the relationships among blood nutrients (amino acids, fatty acids, major elements, trace-elements, and vitamins), BC and nutrient intake in a population of 176 healthy male and female Italian adults between the ages of 65 and 79 years. 89 blood markers, 77 BC parameters and dietary intake were evaluated. Multivariate data analysis was applied to infer relationships between datasets. As expected, the major variability between BC and the blood nutrient profile (BNP) observed was related to sex. Aside from clear sex-specific differences in BC, female subjects had higher BNP levels of copper, copper-to-zinc ratio, phosphorous and holotranscobalamin II and lower concentrations of branched-chain amino acids (BCAAs) and proline. Fat mass, percentage of fat mass, percentage of lean mass and the skeletal muscle index (SMI) correlated the most with BNP in both sexes. Our data showed positive correlations in male subjects among ethanolamine, glycine, albumin, and sulfur with SMI, while palmitoleic acid and oleic acid exhibited negative correlations. This differed in female subjects, where SMI was positively associated with albumin, folic acid and sulfur, while CRP, proline and *cis*-8,11,14-eicosatrienoic acid were negatively correlated. We investigated the influence of diet on the observed BNP and BC correlations. Intriguingly, most of the components of the BNP, except for folate, did not exhibit a correlation with nutrient intake data. An understanding of the physiological and biochemical processes underpinning the observed sex-specific correlations between BNP and BC could help in identifying nutritional strategies to manage BC-changes in aging. This would contribute to a deeper understanding of aging-associated nutritional needs with the aim of helping the elderly population to maintain metabolic health.

## Introduction

Fulfilling nutrient needs with diet is crucial to preventing malnutrition and, consequently, to sustaining metabolic health and quality of life. Dietary guidelines have been established by regulatory authorities according to changing nutritional needs throughout life. For example, caloric requirements decline in later life due to a reduced metabolic rate. On the other hand, there is evidence that specific nutrients (e.g., vitamin A, Ca, Mg, and Se) may be increasingly required in the elderly population (Hoffman, 2017). Aging strongly modifies human physiology, with notable changes in muscle mass and function and in fat and bone mass. With increasing age, a progressive loss of skeletal muscle mass and strength occurs, and this muscle atrophy is prevalent in approximately half of the individuals over the age of 80 years (Kalyani et al., 2014). As a result of skeletal muscle mass loss, the basal metabolic rate is reduced by approximately 30% between the ages of 20 and 70 years (Chau et al., 2008). Concomitantly, these changes in skeletal muscle often lead to reduced physical activity, which further decreases energy requirements for elderly individuals. It has also been reported that muscle strength decreases during aging by 16–41% in individuals older than 40 years, and this decline is more pronounced in women than men (Cruz-Jentoft et al., 2010). Overall, age-related physiological changes in body composition (BC) differ between sexes (Kirchengast and Huber, 2009).

An age-related increase and redistribution of adipose tissue in the abdominal area and visceral organs is well-reported and seems to correlate positively with an increased risk of cardio-metabolic disorders (Hunter et al., 2010). Moreover, bone mineral density (BMD) typically decreases with age, due to impaired bone turnover, with increased bone resorption and gradual development of osteoporosis (Hunter and Sambrook, 2000). This aging-altered bone turnover affects both sexes and cannot be assigned solely to estrogen deprivation (Demontiero et al., 2012). Nevertheless, women lose up to 20% of bone mass during the 5–7 years following menopause relative to 0.5–1% per year for men (JafariNasabian et al., 2017). These age-dependent physiological and metabolic changes require an adaptation of nutritional habits to prevent exceeding energy intake, which leads to the accumulation of fat in the body (Shimokata and Kuzuya, 1993).

Aging-associated variations of BC and physiology affect nutritional status and thus adequate dietary nutrient intake and metabolism. In addition, males and females exhibited a distinct metabolite pattern in plasma (Jove et al., 2016). However, there is an urgent need to develop nutritional solutions that are able to mitigate aging-associated malnutrition and to sustain healthy conditions in the elderly population (Wells and Dumbrell, 2006; Pray et al., 2010). Therefore, proper knowledge of the nutritional status in the elderly population is a prerequisite for providing adequate guidance for nutritional intake (Elmadfa and Meyer, 2014). Epidemiology provides a large amount of dietary habit data, often using self-reported dietary assessments, which sometimes lack accuracy, mainly due to participant misreporting (Archer et al., 2013; Elmadfa and Meyer, 2014). The information collected from self-reported dietary questionnaires is often complemented by the additional analysis of BC and of nutritional markers in biological matrices such as blood and urine. These nutritional markers can encompass indications about specific nutrient status (e.g., estimation of body stores) and function (e.g., assessment of related metabolic function). However, nutrient analysis continues to use relatively classic methodologies, which are often applied to determine a single or limited number of entities at once (Sauberlich, 1999). Today, novel approaches derived from metabolomics lead to new opportunities to acquire a broad range of nutritional status information, i.e., the nutritional phenotype, using a minimum number of analytical procedures (Buescher et al., 2010; Rezzi et al., 2013; Guiraud et al., 2017; Konz et al., 2017; Petruzziello et al., 2017). In contrast to classical single-nutrient-oriented methods, the nutritional phenotyping approach is based on the integration of multiple complementary analytical platforms. The quantification of minerals/elements, fatty acids, amino acids, and vitamins is essentially based on mass spectrometry. Indeed, an increasing number of studies demonstrate the relevance of mass spectrometry-based nutrient analysis to identify single nutrients or patterns of nutrients associated with lean mass or cognitive decline in the elderly (Ispoglou et al., 2016; Oulhaj et al., 2016).

However, there are a limited number of studies reporting associations between limited sets of blood nutrients and BC parameters (B) (Mahabir et al., 2008; Jourdan et al., 2012). This is, to the best of our knowledge, the first time that comprehensive datasets of blood nutrients (namely, amino acids, fatty acids, minerals, and vitamins), dietary intakes and BC parameters were evaluated for possible associations. Multivariate data analysis was used to decipher a relationship between sex and BC-specific BNP signatures. Hereafter, we discuss the possible biological and nutritional implications, considering the dietary nutrient intake data (obtained from a seven-day food records including mineral/vitamin supplements). The obtained data shed new light on the interplay among sex, BC, nutrition, and BNP in the elderly.

## Materials and Methods

### Clinical Study

The study was conducted in collaboration with the EU-NUAGE consortium (Santoro et al., 2014) and Bologna University, Italy, and aimed to define new stratification tools and identify specific nutritional requirements in fully characterized elderly subjects. A total of 176 healthy and free-living adults (age range 65–79, 83 males and 93 females) were selected from the Italian cohort based upon the availability of blood samples. Human serum and plasma samples were collected on fasting individuals usually on study day eight, just after the completion of the assessment of dietary intakes (seven-day food records). The main characteristics of the study population are shown in Table 1. The clinical protocol was approved by the Independent Ethics Committee of the Sant’Orsola-Malpighi Hospital, Bologna, Italy, and Ethics Committee, Canton of Vaud, Switzerland. All subjects gave written informed consent in accordance with the Declaration of Helsinki. The use of a human plasma pool and serum reference material for amino acid and mineral analysis, respectively, was approved by the Ethics Committee, Canton of Vaud, Switzerland.

**Table 1:** Population characteristics.

Variable	Males (*n* = 83) Median (minimum, maximum)	Females (*n* = 93) Median (minimum, maximum)	*p*-Value
Age (years)	73 (65, 79)	72 (65, 79)	0.44
BMI (kg/m^2^)	27.3 (18.7, 41.6)	26.1 (18.8, 44.6)	0.30
Height (m)	1.72 (1.49, 1.87)	1.59 (1.13, 1.77)	5.23E-24
Waist (cm)	97.7 (78.0, 125.5)	87.2 (65.0, 120.0)	4.08E-11
Hip (cm)	101.5 (84.5, 121.0)	101.0 (86.0, 128.2)	0.33
ALMI (kg/m^2^)	8.22 (6.45, 10.96)	6.45 (4.88, 11.20)	5.62E-24
LMI (kg/m^2^)	17.83 (15.11, 24.57)	14.92 (12.03, 25.28)	1.46E-21
SMI	0.30 (0.26, 0.36)	0.25 (0.18, 0.32)	5.05E-29
FMI (kg/m^2^)	8.71 (2.85, 16.80)	10.86 (3.92, 18.90)	2.54E-07
Whole-body weight (kg)	80.551 (55.02, 112.15)	65.8 (45.79, 98.07)	2.39E-14
Percentage of fat mass	31.91 (14.78, 42.77)	40.56 (20.71, 55.43)	1.97E-18
Percentage of lean mass	64.64 (54.74, 81.04)	56.47 (42.05, 75.66)	5.81E-18
Whole-body BMD	1.16 (0.89, 1.41)	0.96 (0.68, 1.33)	9.24E-26


*BMI, body mass index; ALMI, appendicular lean mass index; LMI, lean mass index; SMI, skeletal muscle mass index; FMI, fat mass index; BMD, bone mineral density.*

### Seven-Day Food Records and Physical Activity

Seven-day food records as described elsewhere (Ostan et al., 2018) were used to assess dietary intake. Briefly, participants received exhaustive instructions by a trained interviewer before completing the questionnaire. A recommendation to collect data at the time of food consumption and not change eating habits during the week of registration followed. At the end of the recorded period, in-depth interviews were conducted by a dietician/research nutritionist to verify the types and quantity of foods reported. Information such as dressings, preparation methods and recipes as well as an assessment of possible consumption of forgotten foods was obtained. Consumed foods were coded according to standardized coding procedures, and nutrients were then derived from the consumed food using WinFood^®^ software, which uses INRAN (National Institute for Research on Food and Nutrition, Italy) and IEO (European Institute of Oncology, Italy) food composition tables. The daily total intakes of B vitamins (B1, B2, B3, B5, B6, B8, B9, and B12), vitamin A, vitamin C, vitamin D, vitamin E, vitamin K, calcium, chromium, iron, phosphorus, iodine, magnesium, manganese, copper, selenium, zinc, molybdenum, potassium, and omega 3 fatty acids (*cis*-5,8,11,14,17-eicosapentaenoic acid and *cis*-4,7,10,13,16,19-docosahexaenoic acid) were obtained. The dietary intakes from the seven-day food records were added/summed to the intakes of related dietary supplements as assessed by a specific vitamin/mineral supplements questionnaire. Physical activity was assessed by using the physical activity scale for the elderly (PASE). Therefore, a questionnaire was completed with the participants and a trained interviewer during the general interview.

### Body Composition Data

A whole-body dual energy X-ray absorptiometry (DXA) scan was performed to determine total and regional BC (Bazzocchi et al., 2016) using a fan-beam densitometer (Lunar iDXA, Madison, WI, United States; enCORETM 2011 software version 13.6). Participants were placed in a supine position with arms at their sides slightly separated from the trunk and correctly centered on the scanning field. Regions of interest were defined by the analytical program and included six different corporeal districts: total body, trunk, upper limbs, lower limbs, android region (a portion of the abdomen included between the line joining the two superior iliac crests and extended cranially up to 20% of the distance between this line and the chin), and the gynoid region (portion of legs from the femoral great trochanter, directed caudally up to a distance double that of the android region). For each region, DXA determined the weight (in g) of total mass, fat mass, lean mass, and bone mineral content (BMC). A total of 77 BC variables, including whole-body and region-specific measurements of weight, fat mass, lean mass, and ratios thereof as well as soft tissue, BMC, BMD, T-score, android-gynoid ratio, and the calculated indexes, including the appendicular lean mass to body weight ratio [i.e., skeletal muscle mass index (SMI)], appendicular lean mass index (ALMI), lean mass index (LMI) and fat mass index (FMI), were determined. SMI is the ratio of arm and leg lean mass divided by the weight of the individual while ALMI refers to the sum of arm and leg lean mass divided by the square of the height (kg m^-2^). The FMI and LMI indexes refer to the whole-body fat mass divided by the square of the height (kg m^-2^) and to the whole-body lean mass divided by the square of the height (kg m^-2^), respectively.

### Instrumentation and Analytical Methods

In-house developed analytical methods were used for the determination of blood nutrient profiles (BNPs), including amino acids, fatty acids and minerals, validated according to internal guidelines covering the assessment of selectivity, linearity of calibration, limit of detection/quantification, robustness, precision, trueness/recovery, measurement uncertainty, carry over and matrix effects (if applicable). To reduce the risk of batch effects, the specimens were randomized according to age and sex. A total of 89 variables, including concentrations of 34 amino acids and related metabolites, including the global arginine bioavailability ratio (GABR), i.e., arginine/(ornithine+citrulline), 28 fatty acids, 19 minerals, 4 vitamins, and 4 additional blood markers [albumin, C-reactive protein (CRP), soluble transferrin receptor and homocysteine], were determined.

#### Amino Acids and Related Metabolites

The analysis of amino acids in human plasma was performed using an Acquity UPLC I Class system coupled to a TQS XEVO triple quadrupole from Waters (Milford, MA, United States). The following were measured: alanine, β-alanine, α-aminobutyric acid, β-aminoisobutyric acid, γ-aminobutyric acid, arginine, asparagine, aspartic acid, citrulline, asymmetric-dimethylarginine, symmetric-dimethylarginine, ethanolamine, glutamic acid, glutamine, glycine, histidine, hydroxyproline, isoleucine, leucine, lysine, methionine, 1-methylhistidine, 3-methylhistidine, ornithine, phenylalanine, proline, sarcosine, serine, taurine, threonine, tryptophan, tyrosine, and valine. Quantification was achieved using an internal standard and an external calibration curve.

### Fatty Acids

The determination of fatty acids in human plasma was performed with the Agilent 7890A capillary fast gas chromatography (GC) system (Agilent Technologies, Santa Clara, CA, United States) equipped with a flame ionization detector (FID). The following were measured: capric acid, lauric acid, myristic acid, myristoleic acid, pentadecanoic acid, palmitic acid, palmitoleic acid, heptadecanoic acid, *cis*-10-heptadecenoic acid, stearic acid, elaidic acid, oleic acid, oleic acid isomer (n-9 *trans*), linoelaidic acid, linoleic acid, gamma-linolenic acid, alpha-linolenic acid, arachidic acid, *cis*-11-eicosenoic acid, *cis*-11,14-eicosadienoic acid, *cis*-8,11,14-eicosatrienoic acid, arachidonic acid, behenic acid, erucic acid, *cis*-5,8,11,14,17-eicosapentaenoic acid (EPA), lignoceric acid, nervonic acid, and *cis-*4,7,10,13,16,19-docosahexaenoic acid (DHA). The quantification of fatty acids was achieved using an internal standard and a response factor (RF) that was evaluated for each fatty acid during method development.

#### Minerals

The determination of minerals was performed using an Agilent 8800 triple quadrupole ICP-MS (Agilent Technologies, Tokyo, Japan) operated in low matrix plasma mode. For the quantification of magnesium (Mg), phosphorous (P), sulfur (S), potassium (K), calcium (Ca), manganese (Mn), iron (Fe), cobalt (Co), copper (Cu), zinc (Zn), selenium (Se), rubidium (Rb), strontium (Sr), molybdenum (Mo) and iodine (I), the external calibration approach was applied and a certified serum reference material was analyzed on a daily basis for quality control (Konz et al., 2017). Standard clinical routine analysis of sodium (Na), chlorine (Cl), and potassium (K) was performed using an Architect plus ci4100 platform from Abbott Laboratories (Lake Bluff, IL, United States) equipped with an electrochemistry module. Individual quality control samples were tested on a daily basis prior to analysis.

#### Vitamins and Blood Markers

Holotranscobalamin II (physiologically active B12), total vitamin B12 and 25-hydroxy vitamin D were determined using an Architect plus ci4100 platform from Abbott Laboratories (Lake Bluff, IL, United States) consisting of a chemistry and immunoassay module. Individual quality control samples were tested on a daily basis prior to analysis. Serum folates were determined using a chemiluminescence assay using an ADVIA Centaur XP immunoassay system (Siemens Healthcare, Erlangen, Germany). Plasma albumin was analyzed using the VITROS ALB slides (Ortho-Clinical Diagnostics, United Kingdom) on a VITROS 5.1/FS analyzer. Plasma hsCRP was measured by ProcartaPlexTM immunoassay (eBioscience, Hatfield, United Kingdom) according to the manufacturer’s instructions. Analysis was performed using Luminex 200 instrumentation (Luminex Corporation, Netherlands). Plasma homocysteine was measured by an enzymatic assay using an Olympus AU400 clinical chemistry platform (Beckman Coulter, High Wycombe, United Kingdom). Serum glucose and serum insulin were determined by biochemical assay and chemiluminescent immunoassay, respectively. Insulin resistance status was calculated according to the homeostasis model assessment of insulin resistance (HOMA-IR) using the following formula: insulin (mIU mL^-1^) x glucose (mmol L^-1^)/22.5 (Matthews et al., 1985).

### Instrumentation and Analytical Methods

All solutions were prepared with 18 MΩ cm^-1^ deionized water obtained from a Milli-Q system (Millipore, Bedford, MA, United States). All chemical substances used for analysis were of the highest grade and purity available. Frozen human serum and plasma samples were thawed and vortexed prior to sample preparation.

Mobile phases for amino acid determination were prepared by 10-fold dilution of AccQ Tag Ultra Eluent A concentrate (10% acetonitrile, 6% formic acid, and 84% ammonium formate in H_2_O) from Waters in H_2_O and AccQ Tag Ultra Eluent B (98% acetonitrile, 2% formic acid), that was applied without dilution, from the same manufacturer. An internal standard solution consisting of labeled amino acids (Cambridge Isotope Laboratories, Tewksbury, MA, United States) was prepared in 0.1 M HCl (Sigma-Aldrich). The calibrants used for external calibration consisted of amino acid standards (Sigma-Aldrich) in 0.1 M HCl. A pool of human plasma (Biopredic International, Saint-Grégoire, France) spiked with known concentrations of all analytes was used for quality control. A derivatization step based on the AccQ Tag Ultra Method from Waters was performed prior to analysis to enhance the sensitivity of the method (see Supplementary Material).

The preparation of fatty acid methyl esters (FAMEs) by transesterification was performed by adding 200 μL of plasma immediately after blood centrifugation in an appropriate vial and mixing the fluid with 200 μL of ethanol (Merck), 100 μL of internal standard TAG 13:0 solution and 100 μL of internal standard FAME 21:0 solution. A mixture containing 2 mL of methanol (Merck), 2 mL of 3N methanol/HCl (Merck) and 1 mL of hexane (Merck) was added, and the tubes were sealed, shaken vigorously and maintained at 100°C for 60 min. After cooling to room temperature, 2 mL of H_2_O was added, followed by centrifugation (1200 × *g*, 5 min), and the upper phase (hexane) was transferred into appropriate glass vials. The two internal standard solutions, TAG 13:0 and FAME 21:0, consisted of 0.1 mg mL^-1^ tritridecanoin (Nu-Chek Prep., Inc., United States, cat # T-135) and 1 mg mL^-1^ of methyl heneicosanoate (Nu-Chek Prep., Inc., United States, cat # N21-M) in 100 mL n-hexane, respectively. The FAME calibration standard mixture GC Standard Nestlé 36 was provided by Nu-Chek Prep., Inc. (Elysian, MN, United States). A total of 100 mg of the standard was dissolved in 200 mL n-hexane to yield a final concentration of 0.5 mg mL^-1^. The calibration standard solution was injected three times every 2 weeks to assess method reproducibility.

Human serum reference material (Seronorm, Trace Elements Serum level II) was obtained from Sero (Billingstad, Norway) and utilized as a quality control for mineral analysis. The lyophilized powder was dissolved in 3 mL H_2_O. Subsequently, human serum samples and reference material were diluted (1:10) using diluent solution. The diluent solution consisted of 5% 1-butanol (99.9%, Sigma-Aldrich, St. Louis, MO, United States), 0.05% EDTA (ethylenediaminetetraacetic acid, 99.995% trace metals basis, Sigma-Aldrich), 0.05% Triton X-100 (BioXtra, Sigma-Aldrich) and 0.25% ammonium hydroxide (Sigma-Aldrich). Calibrants and the online-internal standard were prepared using ICP standards (Merck Millipore, Darmstadt, Germany) in a diluent solution. The tuning solution was purchased from Agilent Technologies (Santa Clara, CA, United States). Instrumental settings for amino acid, fatty acid and mineral analysis are represented in greater detail in the Supplemental Material Section.

### Statistical Analysis

Chemometric analysis was performed on BNP, BC and nutrient intake data using the SIMCA-P+ software package (version 14.0, Umetrics AB, Umeå, Sweden) using log scale transformation whenever variables were not normally distributed. Principal component analysis (PCA) was first employed to explore the variance within the dataset, identify outliers, and assess major confounders related to sex, age, and BMI (Wold et al., 1987a). Data were visualized by means of principal component scores, where each point represents an individual BNP or BC profile of a sample. Variables influencing sample distribution in the PCA space were identified from the corresponding loadings plot. Furthermore, we used supervised multivariate data analysis to display variables with the highest discriminative value between sexes and statistically significant associations between BNP and BC. For this purpose, partial least squares regression analysis (PLSR) and its modification, orthogonal projection to latent structures (OPLS), were employed (Wold et al., 1987b; Trygg and Wold, 2003). Compared to PLS, OPLS models provide sparser models (improving their interpretability) with the same degree of fit as PLSR models. To highlight the weight of individual variables in the model, variable importance in projection (VIP) was used, with a value above 1.5 used as a threshold by convention. In addition, a Pearson correlation coefficient with a *p*-value significant at the 99% confidence interval (e.g., with *N* = 83, *r* > = 0.285, *p* < 0.01) was applied. All graphs and boxplots were prepared using GraphPad prism software.

## Results

### Sexual Dimorphism Is Reflected in the Body Composition

The population demographics of the studied population are shown in Table 1. As expected, there was major sexual dimorphism in BC, such as body height, weight, hip size, and lean- and fat-mass-related BC parameters. Multivariate data analysis was performed on the DXA-derived data using PCA. This multivariate data analysis explored the variance in the data that may explain differences between subject groups. Data were visualized by means of principal component (PC) scores, where each point represents an individual metabolic profile. Variables (e.g., body composition) responsible for the differences between samples can be extracted from the corresponding loadings plot, where each coordinate represents a single variable. The distribution of the subjects based on their BC was predominantly driven by sex (explaining 79.7% of the data variance in the first two PCs; data not shown). Females were characterized by a higher fat mass at both the regional (upper and lower limbs) and whole-body level. Males had higher values of whole-body and region-specific BMC, lean mass, BMD, weight, soft tissue, T-score, and ALMI and LMI.

### Dietary Intakes

Seven-day food records were used to assess dietary intakes. The results were normalized to the body weight of the individuals, facilitating a comparison between males and females. The dietary intakes of nutrients for males and females are shown in Table 2. As seen, the daily mean dietary intakes of water, folic acid, vitamin B2, vitamin B3, vitamin A, vitamin C, vitamin D, and calcium were significantly higher in females compared to males. No significant differences were observed for total dietary intake of energy, carbohydrates, saturated and unsaturated fatty acids and protein intake, normalized by body weight (see Table 2). In Supplementary Table 1, the mean daily dietary intakes (normalized to body weight) of different food groups were compared for both sexes. The intake of white grains and alcohol were significantly higher in men than in women (*p* = 0.04 and *p* = 0.001, respectively) while the dietary intake of whole grains, fruits, vegetables, legumes, dairy, cheese, meat, and sugars were not significantly different between males and females.

**Table 2:** Comparison of mean daily intake (values normalized to body weight) of energy and nutrients between men and women.

	Normalized to whole-body weight (kg)				
	
	Male (*n* = 83)		Female (*n* = 92)		
			
	Mean^a^	*SD*	Mean^a^	*SD*	*p^b^*
Total energy (kcal)	24.28	6.06	24.49	6.26	0.858
Total carbohydrates (g)	3.12	0.86	3.20	1.02	0.936
Total fats (g)	0.85	0.26	0.88	0.23	0.273
Total saturated fatty acids (g)	0.26	0.08	0.28	0.08	0.218
Total MUFA^c^ (g)	0.38	0.13	0.40	0.12	0.446
Total PUFA^d^ (g)	0.12	0.06	0.12	0.05	0.441
ω-3 PUFA (g)	0.01	0.01	0.01	0.01	0.557
ω-6 PUFA (g)	0.07	0.04	0.07	0.03	0.376
Cholesterol (mg)	2.77	0.90	2.92	1.03	0.471
Total proteins (g)	0.93	0.23	0.97	0.23	0.196
Animal proteins (g)	0.44	0.15	0.47	0.14	0.182
Vegetal proteins (g)	0.35	0.14	0.35	0.15	0.432
Total dietary fiber (g)	0.30	0.13	0.33	0.15	0.368
Starch (g)	1.38	0.53	1.43	0.58	0.609
Water (g)	24.13	8.80	29.09	9.98	0.001
Biotin (mg)	0.25	0.16	0.31	0.26	0.197
Folic acid (μg)	3.72	1.67	4.76	2.23	0.003
β-carotene (μg)	23.15	15.93	26.72	20.67	0.396
Vitamin B1 (mg)	0.03	0.13	0.02	0.01	0.296
Vitamin B2 (mg)	0.02	0.01	0.04	0.10	0.013
Vitamin B3 (mg)	0.25	0.10	0.31	0.19	0.018
Vitamin B5 (mg)	0.03	0.02	0.04	0.03	0.148
Vitamin B6 (mg)	0.04	0.19	0.03	0.01	0.061
Vitamin B12 (μg)	0.13	0.64	0.06	0.08	0.793
Vitamin A (μg)	12.99	10.34	16.08	12.57	0.029
Vitamin C (mg)	1.86	1.56	2.11	1.26	0.020
Vitamin D (μg)	0.05	0.10	0.26	0.84	<0.001
Vitamin E (mg)	0.20	0.58	0.15	0.08	0.367
Calcium (mg)	9.94	3.96	13.10	6.52	<0.001
Copper (mg)	0.02	0.01	0.11	0.86	0.535
Iron (mg)	0.17	0.10	0.17	0.08	0.858
Iodine (μg)	1.63	0.79	1.78	0.77	0.083
Potassium (mg)	38.80	12.20	40.44	12.65	0.471
Magnesium (mg)	3.37	1.24	3.87	1.65	0.055
Manganese (mg)	0.02	0.01	0.02	0.02	0.518
Phosphorus (mg)	16.58	5.16	17.75	6.96	0.443
Selenium (μg)	0.52	0.28	0.58	0.34	0.268
Sodium (mg)	24.07	8.80	24.83	8.42	0.507
Zinc (mg)	0.13	0.08	0.14	0.05	0.073


*^*a*^Mean daily dietary intake normalized to body weight (kg); ^*b*^Mann–Whitney *U*-test; ^*c*^Monounsaturated fatty acids; ^*d*^Polyunsaturated fatty acids.*

### Analysis of BNP Reveals a Sex-Specific Signature

Similarly, the application of PCA to BNP and blood marker data revealed sex as a major factor contributing to the explained variance in the first three PCs (38.8% of the total variance; data not shown).

**FIGURE 1:**
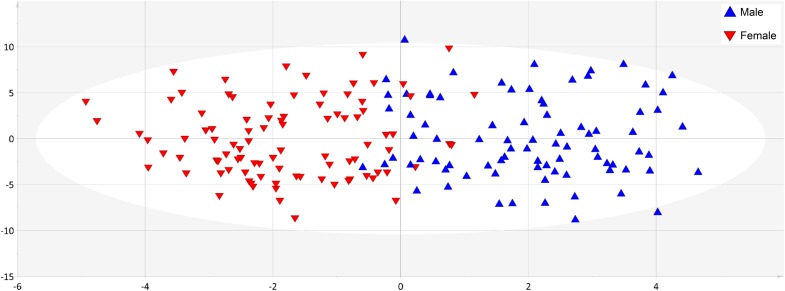
Blood nutrient dataset showed clear separation of sexes. The application of orthogonal corrected partial least squares discriminant analysis (OPLS-DA) to the data set exhibited good predictability of sex from BNP values, as indicated by a cumulative Q^2^Y value of 0.579. Blue triangles correspond to male and red triangles to female individuals.

**FIGURE 2:**
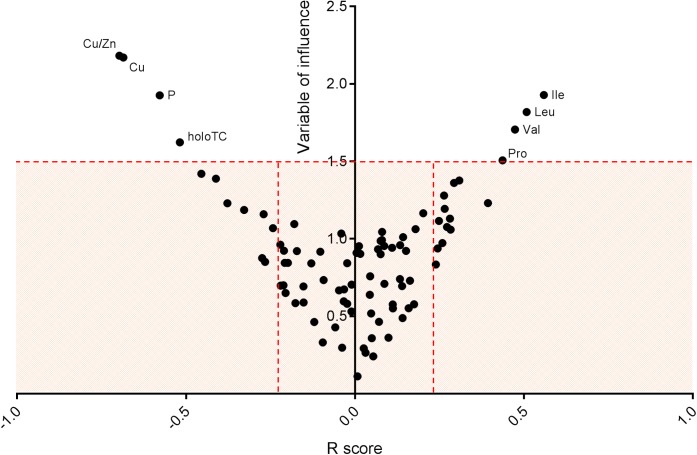
Significant blood nutrient parameters that contribute to sex differences. Representation of variable of influence (VIP) vs. R score. The V-plot was applied to identify the most significant variables (BNP) contributing to the differences driven by sex. Statistically significant variables at the 99% confidence interval were selected using a VIP threshold of 1.5 and *r* ≥ 0.2. Female BNP were marked by higher values of Cu, Cu/Zn, P, and holotranscobalamin II (active B12) and lower concentrations of isoleucine, leucine, valine, proline and, to a minor degree, homocysteine.

**FIGURE 3:**
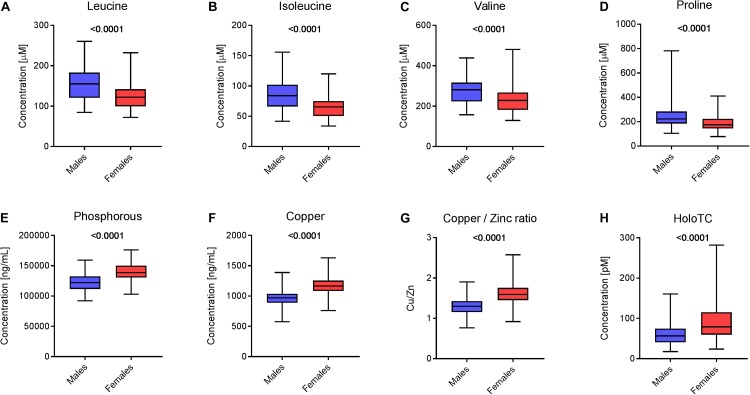
Sex-specific differences in blood nutrients. Box plots representing the concentration of selected blood nutrients in male and female populations (*N* = 83 males and 93 females). Statistics with an unpaired Student’s *t*-test (*p*-value is depicted above the box plot). Concentration of **(A)** leucine, **(B)** isoleucine, **(C)** valine, **(D)** proline, **(E)** phosphorus, **(F)** copper, **(G)** the ratio between the concentrations of copper and zinc and **(H**) holotranscobalamin II in the two populations.

Supervised methods such as OPLS discriminant analysis were applied to maximize the discrimination of groups according to sex (Figure 1). The modeling of the sex-related differences was robust (as indicated by a Q^2^Y value of 0.579). Using a VIP value above 1.5 and a Pearson correlation coefficient with a *p*-value significant at the 99% confidence interval, the most statistically significant variables driving the observed differences among the sexes are reported in Figure 2. Applying the selected threshold for significance, Figure 2 shows that the female BNPs were marked by higher Cu, copper-to-zinc ratio, P, and holotranscobalamin II (physiologically active B12) values and lower concentrations of isoleucine, leucine, valine, proline and, to a minor degree, homocysteine (Figure 3). Among the proteinogenic amino acids, the strongest differences between sexes were observed for the BCAAs leucine, isoleucine, and valine. The total concentration of BCAAs (sum of isoleucine, leucine, and valine) exhibited positive correlations with the HOMA-IR score for male and female subjects (*r* = 0.34, *p* = 0.0018 and 0.44, *p* < 0.001, respectively). Overall, the mean HOMA-IR values were slightly higher for males compared to females in our cohort (HOMA-IR 2.84 and 2.41, respectively). Phosphorous, copper and the copper-to-zinc ratio were marked by significantly higher values in females. Similarly, holotranscobalamin II was found to be elevated in females, while proline was lower in females compared to males. It is worth mentioning that differences were also observed for the unsaturated fatty acids palmitoleic acid, γ-linolenic acid, and nervonic acid (higher in the blood of females) but were below the selected VIP threshold (data not shown).

### Relationships Between BNP and BC Parameters in Males and Females

**FIGURE 4:**
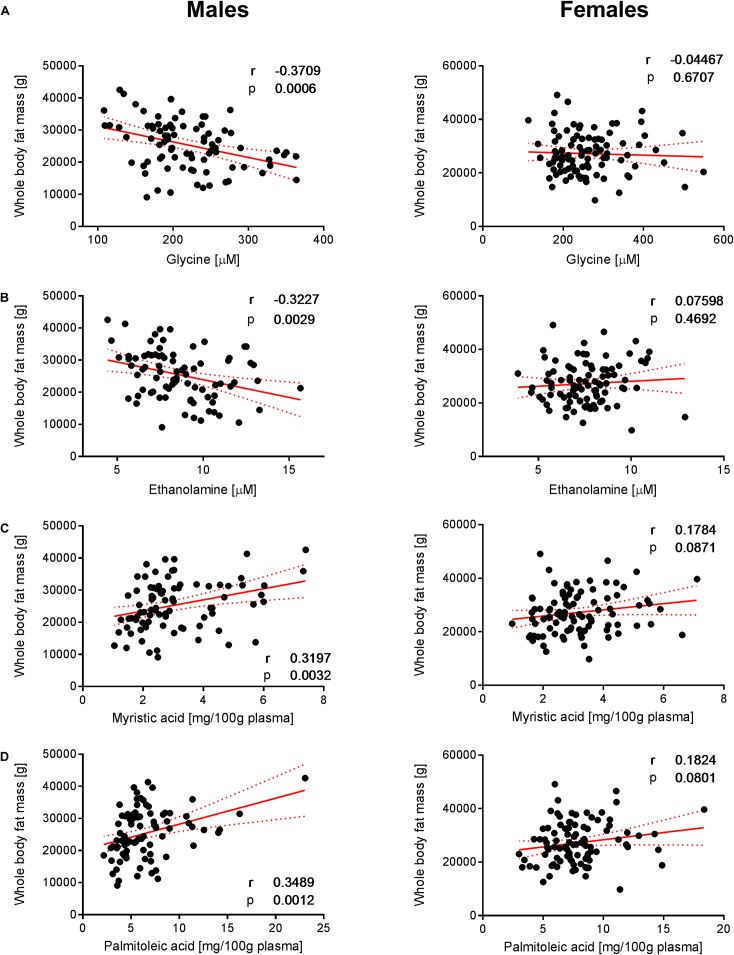
Correlation of glycine, ethanolamine, myristic acid and palmitoleic acid with whole- body fat mass. Significant correlations of **(A)** glycine, **(B)** ethanolamine, **(C)** myristic acid, and **(D)** palmitoleic acid with whole-body fat mass were observed in males but not in females. Pearson’s correlations (r) as well as two-tailed *p*-values are shown. *N* = 83 (males) and *N* = 93 (females). Each dot represents an individual sample. Red dotted lines show the 95% confidence interval.

**FIGURE 5:**
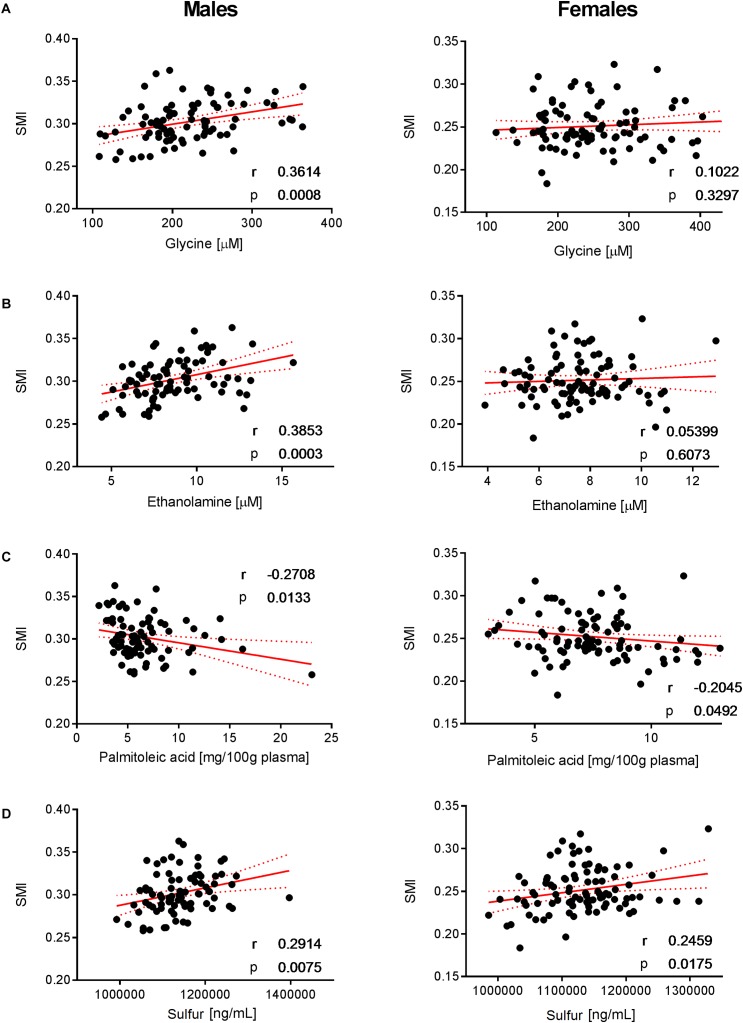
Correlation of glycine, ethanolamine, palmitoleic acid, and SMI. Significant correlations of **(A)** glycine, **(B)** ethanolamine, **(C)** palmitoleic acid, and **(D)** sulfur with SMI were observed in males but not in females. Pearson’s correlations (r) as well as two-tailed *p*-values are shown. *N* = 83 (males) and *N* = 93 (females). Each dot represents an individual sample. Red dotted lines show 95% confidence interval.

**FIGURE 6:**
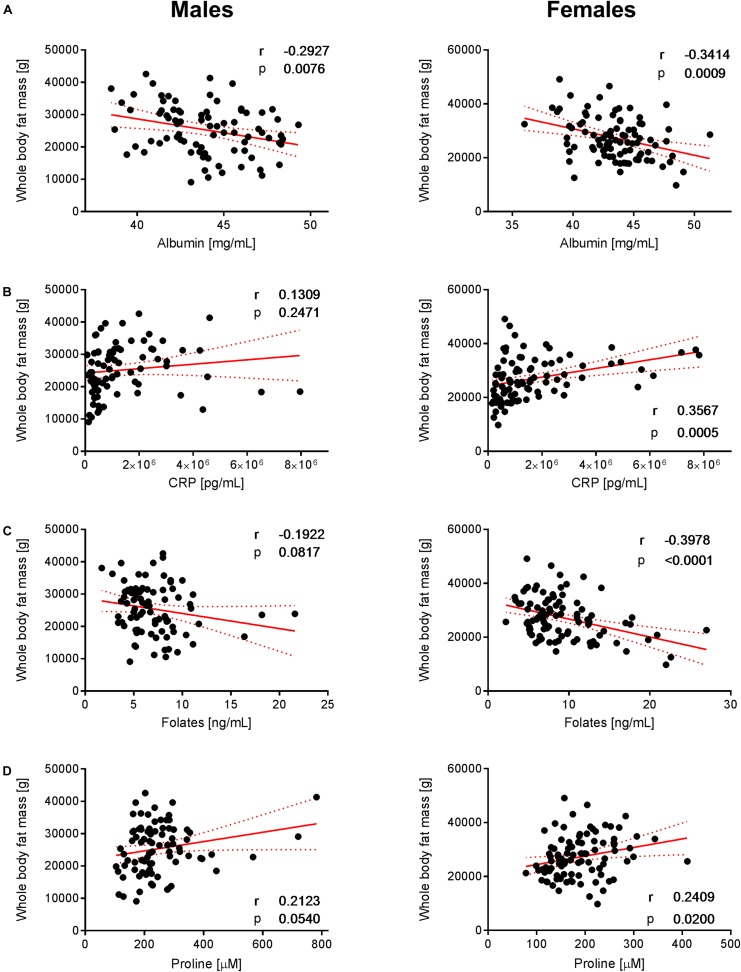
Correlation of albumin, CRP, folates, and proline with whole-body fat mass. The data set showed significant correlations of **(A)** albumin, **(B)** CRP, **(C)** folates, and **(D)** proline with whole-body fat mass in females but not in males (with the exception of albumin that exhibits a negative correlation with whole-body fat mass in both sexes). Pearson’s correlations (r) as well as two-tailed *p*-value are shown. *N* = 83 (males) and *N* = 93 (females). Each dot represents an individual sample. Red dotted lines show the 95% confidence interval.

**FIGURE 7:**
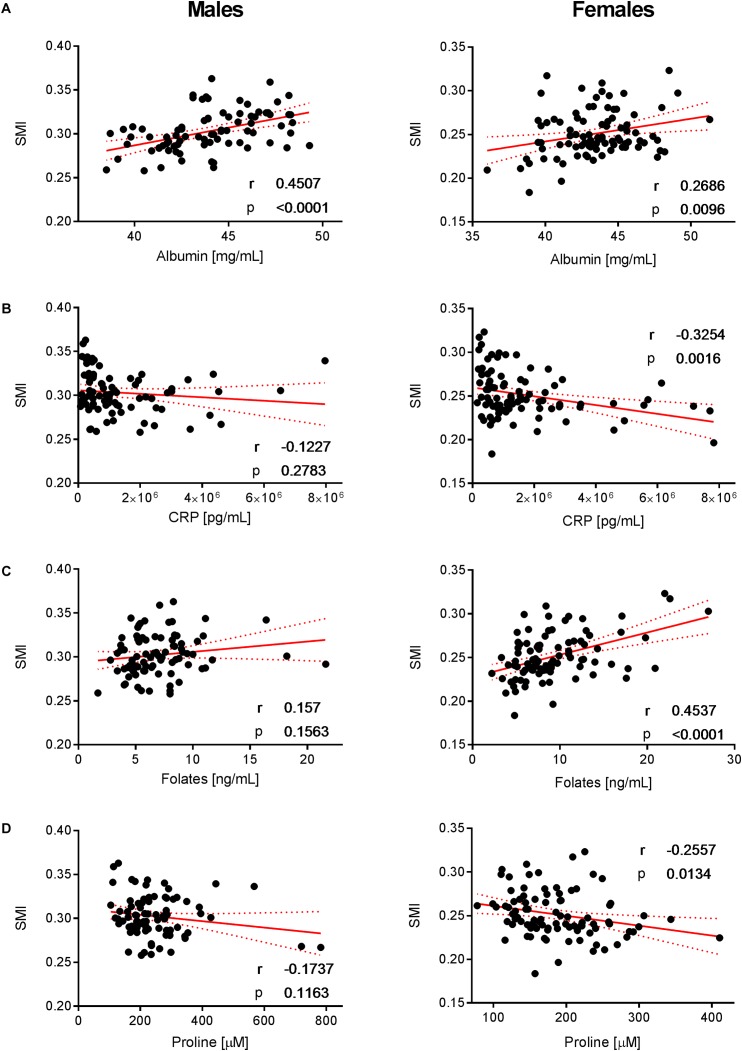
Correlation of albumin, CRP, folates, and proline with SMI. Significant correlations of **(A)** albumin, **(B)** CRP, **(C)** folates, and **(D)** proline with SMI in females but not in males (with the exception of albumin that exhibits a positive correlation with SMI in both sexes). Pearson’ correlations (r) as well as two-tailed *p*-values are shown. *N* = 83 (males) and *N* = 93 (females). Each dot represents an individual sample. Red dotted lines show the 95% confidence interval.

To decipher the effect of sex, we further explored the variability of BNP and its relationships with BC for male and female subjects separately. PCA captured 40.6% and 39.2% of the BNP total variance on the first three PCs for male and female subjects, respectively (data not shown). For both sexes, the blood levels of amino acids and related metabolites contributed to the distribution of BNP along the first PC. The OPLS was applied to explore statistically significant associations between BNP and BC parameters in males and females. Because of the inherent relationships between measurements in the DXA dataset (e.g., strongly correlated variables such as total and regional fat mass), we selected a total of eight BC parameters to build OPLS models. The parameters selected were whole-body weight, whole-body fat mass, whole-body percentage of fat mass, whole-body percentage of lean mass, FMI, SMI, android fat mass and gynoid fat mass. This BC parameter selection considered both their physiological relevance for the purpose of this study and statistical validity criteria (goodness of fit R^2^Y, predictability Q^2^Y above 0.2) (Table 3). The exploration of the loadings plot for each OPLS model enabled the identification of statistically significant correlations between BNP and BC parameters (Table 4). Most of the BNP variables identified by the OPLS models were recurrent across the tested BC parameters within each sex, and this likely resulted from the interdependency (e.g., correlation) between the BC parameters. The OPLS models created for male individuals revealed that the circulating levels of ethanolamine, glycine, myristic acid, and palmitoleic acid represent the main and recurrent components of the BNP signature of the BC. Figures 4, 5 display the correlations of these nutrients with whole-body fat mass and SMI, respectively. Additional amino acids (glutamine, glutamic acid, and serine), minerals (Mg, Cu, and S), fatty acids (palmitic, oleic, n-9 *trans* oleic, linoleic, linoelaidic, and γ-linolenic acids) and proteins (CRP and albumin) appeared statistically significant for specific BC parameters in males (see Table 4). On the other hand, circulating concentrations of albumin, folic acid, CRP and proline consistently appeared as a BNP signature of BC parameters in female subjects, as shown in Figures 6, 7 for whole-body fat mass and SMI, respectively. Other molecules, including amino acids (isoleucine, valine), minerals (S, Se), and fatty acids (myristoleic, palmitoleic, and *cis*-8,11,14-eicosatrienoic acids), show a statistically significant correlation with the specific BC parameter (Table 4).

**Table 3:** Summary of statistical characteristics of BC and BNP OPLS models.

	Male subjects			Female subjects		
		
BC parameter	R^2^X (cum)	R^2^Y (cum)	Q^2^Y (cum)	R^2^X (cum)	R^2^Y (cum)	Q^2^Y (cum)
Whole-body weight	0.27	0.55	0.32	0.26	0.43	0.06
Whole-body fat mass	0.28	0.6	0.4	0.26	0.51	0.2
Whole-body percentage of fat mass	0.39	0.64	0.36	0.26	0.57	0.3
Whole-body percentage of lean mass	0.28	0.57	0.35	0.26	0.57	0.3
FMI	0.28	0.58	0.36	0.26	0.47	0.17
SMI	0.27	0.55	0.23	0.24	0.53	0.22
Android fat mass	0.28	0.58	0.37	0.26	0.54	0.25
Gynoid fat mass	0.38	0.66	0.35	0.26	0.44	0.06


*BC, body composition; BNP, blood nutrient profile; OPLSs, orthogonal projection to latent structures; FMI, fat mass index; SMI, skeletal muscle mass index. R^*2*^X: explained variance in the data, R^*2*^Y: explained group variance, Q^*2*^Y: robustness of the model.*

**Table 4:** Blood profiling signatures associated with body composition parameters in male and female subjects.

			Males		Females	
				
BC parameter			Positively correlated	Negatively correlated	Positively correlated	Negatively correlated
1	Whole-body weight	Whole-body level	Myristic acid, palmitoleic acid	Ethanolamine, glycine, Cu, glutamine, serine	CRP, proline, isoleucine, valine	Albumin, folic acid
2	Whole-body fat mass		Myristic acid, palmitoleic acid, oleic acid, linoelaidic acid	Ethanolamine, glycine, albumin	CRP, proline, isoleucine, valine, myristoleic acid	Albumin, folic acid
3	Whole-body percentage of fat mass		Palmitoleic acid, oleic acid	Ethanolamine, glycine, Mg	CRP, proline	Albumin, folic acid
4	FMI		Myristic acid, palmitoleic acid, oleic acid, linoelaidic acid	Ethanolamine, glycine	CRP, proline, myristoleic acid, palmitoleic acid	Folic acid
5	Whole-body percentage of lean mass		Ethanolamine^∗^, glycine^∗^	Myristic acid, palmitic acid, palmitoleic acid, oleic acid, oleic acid isomer (n-9 *trans*), linoelaidic acid, γ-linolenic acid, glutamic acid, CRP	Albumin, folic acid	CRP, proline
6	SMI	Region-specific level	Ethanolamine, glycine, albumin, S	Palmitoleic acid, oleic acid	Albumin, folic acid, S	CRP, proline, *cis-*8,11,14-eicosatrienoic acid
7	Android fat mass		Myristic acid, palmitic acid, palmitoleic acid, oleic acid, oleic acid isomer (n-9 *trans*), linoelaidic acid, glutamic acid	Glycine	CRP, proline, isoleucine, valine, myristoleic acid	Folic acid
8	Gynoid fat mass		Palmitoleic acid	Ethanolamine, glycine, serine	CRP, proline	Albumin, folic acid, S, Se


*BC, body composition; FMI, fat mass index; SMI, skeletal muscle mass index; CRP, C-reactive protein. Variable selection criteria: OPLS Pearson’s correlation coefficient ≥ 0.19 and 0.27 for male (*n* = 83) and female (*n* = 93) subjects, respectively (*p*-value < 0.01); VIP ≥ 1.5. ^∗^1.30 ≤ VIP values ≤ 1.49.*

For both sexes, the OPLS models suggested that the BC-specific BNP signature is mainly driven by whole-body and region-specific fat mass. Among the four BC-derived indexes (FMI, ALMI, LMI, and SMI), only FMI and SMI provided statistically significant OPLS models.

### Relationships Between Daily Nutrient Intake and BC-Specific BNP Signatures

We sought to investigate putative relationships between the molecules of the identified BC-specific BNP signatures (see Table 4) with diet. To achieve this, we computed the OPLS between single molecules from the identified signatures with nutrient intake data (Table 2). Among all tested blood measurements, only folic acid exhibited a statistically significant positive correlation between its estimated intake and blood levels in female subjects (*r* = 0.42, *p* = 0.0001, Supplementary Figure 1).

## Discussion

### Sexual Dimorphism Is Reflected in the Body Composition of Elderly Individuals

Our data were in agreement with the literature and showed significant sexual dimorphism in BCP (body composition parameter) at both the whole-body and region-specific levels, with a higher content of fat and lean mass for females and males, respectively (Kirchengast, 2010). Other BCP contributing to the sexual dimorphism include BMD and the T-score, two correlated measurements showing higher values in male subjects. This observation is in agreement with the known sex-specific bone remodeling with the higher bone resorption rate reported in females (Feng and McDonald, 2011).

### Sexual Imprinting on Specific Blood Nutrients and Influence of Dietary Intake

Similar to BCP, the statistical analysis of the BNP exhibited a good correlation with sex. As pointed out in the results section, a broad set of amino acids, fatty acids, and minerals contributed significantly to the overall dimorphism of BNP. The circulating levels of BCAAs (leucine, isoleucine, valine) and proline were particularly higher in males compared to females (see Figures 3A–C). This finding points toward sex-specific alterations in amino acid metabolism that were previously reported (Krumsiek et al., 2015). This could relate to the development of insulin resistance (Connelly et al., 2017) that is recognized as a metabolic hallmark of age-associated diseases such as type-2-diabetes mellitus and sarcopenia (Taylor, 2012; Cleasby et al., 2016). Moreover, positive relationships between blood BCAA concentrations and insulin resistance were predominantly reported in obese males relative to females (Zhao et al., 2016). In the present study, we also observed positive correlations between BCAAs and the HOMA-IR score for male and female subjects. Moreover, in our cohort, we found a slightly higher mean value for the HOMA-IR in male individuals.

Our data show that the minerals phosphorous and copper and the copper-to-zinc ratio (Cu/Zn) were significantly elevated in females (Figures 3E–G). An increased concentration of circulating phosphorous in female subjects (see Figure 3E) was expected due to the elevated bone resorption rate in elderly women, as discussed above. Bones are the body’s main phosphorous stores. If bone mass decreases, phosphorous is subsequently liberated into circulation. We also observed higher serum Cu and concomitant copper-to-zinc ratios in females, which is in good agreement with previous observations (Konz et al., 2017). The copper-to-zinc ratio has been proposed as a biomarker for health in the elderly population (Malavolta et al., 2010). Indeed, several studies have confirmed the predictive value of an increased copper-to-zinc ratio for unspecific mortality and poor health in elderly subjects (Malavolta et al., 2010; Gaier et al., 2012; Mocchegiani et al., 2012). However, previous studies have failed to explain why the copper-to-zinc ratio is higher in females compared to males. There is evidence that higher serum copper-to-zinc ratios in elderly females are related to inflammaging (Chung et al., 2009). The effects of aging on the human immune system are different for males and females, showing a stronger pro-inflammatory response in females (Marttila et al., 2013). This is in agreement with our data, which showed slightly higher CRP concentrations in females compared to males (median: 1.07 and 0.87 mg L^-1^, respectively). Other studies have even found that the median CRP level was nearly twice as high in female subjects (Khera et al., 2005). Inflammation induces ambivalent changes in several serum metal-binding proteins, which may explain the higher copper-to-zinc ratio in women. In circulation, Cu and Zn are mainly transported by ceruloplasmin (Cp) and human serum albumin (HSA), respectively. Cp is a positive and HSA a negative acute phase reactant. During acute phase response, cytokines are secreted into circulation, suppressing the synthesis of albumin and upregulating the synthesis of Cp (Malavolta et al., 2015). Concomitantly, Zn is displaced from HSA during inflammation and transported into tissues where Zn is required for T-cell production. This compartmentalization limits the bioavailability of Zn to pathogens in circulation and contributes to increased copper-to-zinc ratios in the elderly.

Females also showed higher concentrations of active B12 (holotranscobalamin II) (Figure 3H) and lower plasma concentrations of homocysteine compared to males (data not shown). In circulation, vitamin B12 is partly bound to the transporter transcobalamin. Physiologically active B12 or holotranscobalamin II mediates the transport of the vitamin to tissues (Quadros and Sequeira, 2013). Following cellular uptake, vitamin B12 acts as a cofactor for the enzyme methionine synthase, which plays a crucial role in one-carbon metabolism (Selhub, 2002). The enzyme catalyzes the transfer of a methyl group to homocysteine, forming methionine. Consequently, a decreased pool of vitamin B12 may result in a higher level of homocysteine, which could explain our finding.

It is likely that specific blood nutrients are subject to physical activity and sensory functions (e.g., taste, smell, appetite), which are themselves influenced by sex and could partly explain the observed sexual imprinting. There is clear evidence that sensory abilities such as taste sensitivity and odor identification are impaired in the elderly (Larsson et al., 2000; Yoshinaka et al., 2016). Although some authors report that sex has no effect on the detection or identification of olfactory information (Larsson et al., 2000), most studies showed evidence that the sensory abilities of women are generally better in late life (Yoshinaka et al., 2016; Boesveldt et al., 2017). However, in this study, we have not assessed sensory functions such as taste, smell, or appetite. Likewise, we have inspected the Physical Activity Scale for the Elderly (PASE) score for possible correlations with BC, dietary intake or blood nutrient concentration, but none of these parameters exhibited a significant correlation with the activity score (data not shown).

In this study cohort, differences in dietary intake between male and female elderly were rather limited (Table 2) and poorly correlated with actual blood nutrient levels. Among all blood parameters under evaluation, only folates exhibited a statistically significant positive correlation between their intake and blood levels in females, confirming previous results. Park et al. (2013) reviewed studies on the dietary intake and biological measurement of folate and reported variable positive correlations between 0.05 and 0.54. Overall, this lack of correlation between the datasets could be due to the estimates of nutrient intake values compared to actual quantitative measurements of blood nutrient markers. It is also likely that our investigated cohort (*N* = 176) is limited in its ability to capture enough variable distribution to identify a larger number of significant correlations between datasets. Furthermore, concentrations of blood nutrients can also be determined by individual-specific variations in nutrient metabolism during aging rather than estimated dietary intake values (Houtkooper et al., 2011).

### Sex-Specific Relationships Between Blood Nutrients and Body Composition

To better understand the sex-specific relationships between BC and BNP, the dataset was evaluated for each sex separately by applying OPLS models. Table 4 highlights the blood nutrients, which showed significant relationships with BC according to the generated OPLS models. It is striking that the pattern of blood-based nutrients correlated with BC differed strongly between the two sexes. At the whole-body level, positive correlations existed in males among specific fatty acids (myristic, palmitoleic, oleic, linoelaidic acids) and fat mass (Table 4, lines 1–5). This may arise from an augmented mobilization of lipids from adipose tissues. It has been demonstrated that fatty acids are mobilized from adipose cells that lose the ability to respond to insulin (Karpe et al., 2011). This hypothesis is in agreement with the higher level of BCAAs in males, which has been shown to promote IR (Zhao et al., 2016). Furthermore, negative correlations between the fat-associated BCP and BNP were observed for ethanolamine and glycine in males.

Interestingly, we observed a series of BNPs in females that appear to be statistically significant for specific BC parameters but differ widely from the pattern observed in males. Proline and CRP showed positive and albumin and folic acid negative correlations with whole-body BCP in females (see Table 4, lines 1–5), particularly with fat mass. The OPLS models for lean mass (see Table 4, line 5) revealed that most of the nutrients correlating with fat mass also correlate with lean mass but in different directions. This “mirror-effect” between adipose and muscle tissue became evident in the case of CRP and proline vs. albumin and folic acid (see Table 4). Our data showed similar trends for males, particularly for ethanolamine and glycine, which correlated positively with fat mass but negatively with lean mass. When creating the OPLS models for region-specific BCP, such as the SMI, android and gynoid fat mass, we observed that similar nutritional patterns appear (see Table 4, lines 6–8). As an example, the same nutrients that correlated positively with SMI in males, namely, ethanolamine, glycine and S, correlated also with the whole-body percentage of lean mass, with the exception of S. Moreover, the previously mentioned “mirror-effect” between fat and lean mass was also represented at region-specific levels (Table 4, lines 6–8). Overall, these findings indicate that the assessment of region-specific BCP provides information comparable to whole-body-related BNP.

Ethanolamine is one of the nutrients that showed a strong sex-specific correlation with BCP, particularly with fat and lean mass. This essential micronutrient is a building block for the synthesis of phosphatidylethanolamine (PE). This species accounts for the second most abundant glycerophospholipid group in eukaryotic cells. Phosphatidylethanolamines have a wide range of structural and functional properties in the cell, including the synthesis of other lipids, mitochondrial biogenesis, and autophagy (Calzada et al., 2016). Its homeostasis is controlled through dietary intake and the endogenous metabolism of PE. The CDP-ethanolamine (cytidine diphosphate ethanolamine) or Kennedy pathway represents a major route to PE supply in cells (Vance and Vance, 2004; Rockenfeller et al., 2015). In this pathway, ethanolamine is converted to PE through three sequential enzymatic steps, the last one being controlled by 1,2-diacylglycerol ethanolamine phosphotransferase, which attaches a diacylglycerol unit to CDP-ethanolamine. Our data showed that the ethanolamine blood levels associate positively with SMI and that this association is stronger in male relatively to female subjects (see Figure 5B). Ethanolamine also showed a significant association with fat mass, unlike in female subjects (see Figure 4B). The urinary excretion of ethanolamine was found to be positively associated with BMI in a relatively large cohort of subjects (Elliott et al., 2015). In this study, the authors proposed that ethanolamine may be an intrinsic marker of skeletal muscle turnover. The disruption of CDP-ethanolamine in mouse muscle was achieved by deletion of phosphoethanolamine cytidylyltransferase, the rate-limiting enzyme in this pathway (Selathurai et al., 2015). In this work, the authors reported increased accumulation of intramyocellular and membrane-associated diacylglycerol (DAG) without changing insulin resistance. These results do not support the DAG-induced insulin resistance hypothesis (Samuel et al., 2010), which is considered a metabolic hallmark of aging. However, our results suggest that the higher bioavailability of extracellular ethanolamine could favor PE synthesis through the CDP-ethanolamine pathway and thereby contribute to the control of the intracellular DAG pool. The higher level of blood ethanolamine could also be interpreted as a higher intake of ethanolamine-rich foods and/or a higher turnover of PE metabolism that would be more prevalent in male subjects. However, the analysis of dietary intakes in males and females did not reveal any significant correlation with circulating ethanolamine. On the other hand, the association of the blood ethanolamine level with SMI could also suggest that extracellular ethanolamine would be key to supporting PE recycling, thus contributing to the maintenance of cell membrane composition and functions (Patel and Witt, 2017). Finally, among its many cellular functions, PE also plays a key role in the development of the autophagic process via the necessary lipidation of the autophagy-related protein 8 (Atg8) or microtubule-associated protein 1A/1B light chain (LC3) in yeast and mammalian cells, respectively (Ichimura et al., 2000). The importance of this role of PE is well-exemplified by experiments that have shown that ethanolamine supply increases intracellular PE, autophagic flux in yeast and mammalian cells *in vitro* (Rockenfeller et al., 2015). Interestingly, the authors also report on the extension of the lifespan of yeast, mammalian cells, and flies following ethanolamine administration. Because autophagy is a key mechanism in the recycling of metabolic products and organelles during cellular aging, it would be interesting to unravel the relative contribution of PE metabolism in the autophagy-associated health benefits in humans.

Similar to ethanolamine, glycine correlated with lean and fat mass in males (Figures 4A, 5A). It was demonstrated that glycine can protect muscle mass in times of caloric restriction or cachexia due to its anti-inflammatory properties (Koopman et al., 2017). In fact, the administration of the amino acid reduces systemic inflammation by blocking intracellular Ca accumulation, which in turn reduces the expression of TNF-alpha. There is evidence that glycine modulates muscle inflammation through the same mechanism. Intracellular glycine levels were found to be lower in the elderly, particularly under frailty conditions (Koopman et al., 2017). The authors concluded that this is either due to high tissue demand (exceeding nutritional uptake) or enhanced glycine metabolic breakdown (Koopman et al., 2017). Glycine is also required for the biosynthesis of glutathione, which in turn is involved in the protection against ROS. Consequently, glycine exhibits a protective effect on muscle mass and may explain the positive correlation between muscle mass and glycine concentration that was observed in the male individuals.

For females, our data revealed that folic acid shows a negative correlation with whole-body fat mass, while a positive association with SMI was observed (Figures 6C, 7C, respectively). Folic acid deficiency is associated with muscle dysfunction and a high homocysteine blood concentration in the elderly (Mithal et al., 2013). In particular, lower folic acid intakes were observed in sarcopenic patients, while serum homocysteine levels were increased compared to healthy elderly (Ter Borg et al., 2016). Although not appearing as a VIP variable in the tested statistical models, our data confirm a weak negative relationship between circulating homocysteine and folic acid in the study population (Supplementary Figure 2). In addition, we observed that holotranscobalamin II, the physiologically active fraction of circulating vitamin B12, is higher in females (Figure 3H), while concentrations of homocysteine were lower compared to males. Combined with the observation that low muscle mass and physical frailty are associated with vitamin B12 deficiency (Pannerec et al., 2017), this observation points toward a possible implication of vitamin B12 and the one carbon metabolism in the age-related changes in muscle mass, which could be more prominent in females. Serum folate also predicts grip strength in elderly diabetic subjects (Wee, 2016). Interestingly, these deficiencies appear to be reversible, and folate supplementation can trigger beneficial effects in aged skeletal muscle by increasing blood flow and thereby enhancing oxygen and nutrient delivery to myofibers (Romero et al., 2017). Our data showed a slight positive correlation between SMI and folic acid intake in females (Figure 7C). Taken together, our results support the role of folic acid status in the nutritional management of muscle mass and function in the elderly.

We furthermore identified nutrients that show a consistent but opposite association with body fat mass in females. Positive and negative correlations with BCP derived from adipose tissue were found for CRP and albumin, respectively (Figures 6A,B). In this context, it is interesting that several clinical studies have demonstrated a link between the specific pattern of increased CRP and decreased albumin concentrations with sarcopenia, frailty and vascular and non-vascular mortality in elderly subjects (Clarke et al., 2008; Hubbard et al., 2009; Can et al., 2017).

## Conclusion

Overall, our findings add to the evidence that sex is a significant determinant of the relationship between nutritional phenotyping and BC in the elderly. Although descriptive in essence, our work identifies blood signatures of whole-body fat mass, lean mass and SMI involving several nutrients (amino acids, fatty acids, major elements and trace-elements. Additional work is needed to delineate how these signatures vary in the context of a nutritional intervention, taking into consideration sex-related variations in physical activity, physiology (e.g., cardio-vascular functions), sensory function and behavior in the elderly population. For instance, further studies should investigate whether supplementation of the identified nutrients improves their corresponding nutritional status and can be linked with functional and physiological endpoints such as mobility, cognition, or inflammatory status. Proper integrative data mining approaches are also required to leverage nutritional phenotyping and its relationship to dietary intake. Nevertheless, the reported integrative analysis of nutritional phenotype and BC profiles provides key insights into potential nutritional modulations affecting physiologically important processes in age-related fat/lean mass balance. Overall, this study enhances the knowledge of blood nutrient patterns and their relationship to BC and sex. This points toward aging- and sex-associated nutritional needs and may lead to specific dietary recommendations aiming to maintain metabolic health and quality of life in the elderly population.

## Data Availability

The raw data supporting the conclusions of this manuscript will be made available by the authors, without undue reservation, to any qualified researcher.

## Author Contributions

TK and SR coordinated the writing of the publication and wrote major parts of the paper. AS, LG, CM, and F-PM contributed to the writing of the paper. TK, LG, MG, AB, GB, and AS conducted experiments to determine BNP and BCP and contributed to the interpretation. RO performed analysis and interpretation of the data obtained by seven-day food records and a vitamin/mineral supplements questionnaire. CN performed analyses on CRP levels. FK performed analyses on albumin levels. F-PM and SR performed statistical analyses. AS, JF, and CF designed the study and contributed to interpretation. All authors have given approval to the final version of the manuscript.

## Conflict of Interest Statement

TK, LG, CM, FM, and JF are employees of Nestlé SA. SR and MG were employed by the Nestlé SA. SR is employee of the Swiss Vitamin Institute. The remaining authors declare that the research was conducted in the absence of any commercial or financial relationships that could be construed as a potential conflict of interest.
