# Supplementary material for: Sex-Specific Associations of Blood-Based Nutrient Profiling With Body Composition in the Elderly
Source: Front Physiol. 2019 Jan 24;9:1935. doi: 10.3389/fphys.2018.01935 (PMC6353856; doi:10.3389/fphys.2018.01935)
Supplement: Supplementary file 1 [file Data_Sheet_1.docx]

Supplementary Material

**Sex-specific associations of blood-based nutrient profiling with body composition in the elderly**

**Tobias Konz^1^, Aurelia Santoro^2,3^, Laurence Goulet^1^, Alberto Bazzocchi^4^, Giuseppe Battista^2^, Claudio Nicoletti^5,6^, Fawzi Kadi^7^, Rita Ostan^2,3^, Michael Goy^8^, Caroline Monnard^1^, François-Pierre Martin^8^, Jerome N. Feige^8^, Claudio Franceschi^2,9^, Serge Rezzi^1*,**^**

^1^Nestlé Research, Vers-chez-les-Blanc, Lausanne, Switzerland.

^2^Department of Experimental, Diagnostic and Specialty Medicine, Alma Mater Studiorum, University of Bologna, Bologna, Italy.

^3^C.I.G. Interdepartmental Centre “L. Galvani”, Alma Mater Studiorum, University of Bologna, Bologna, Italy.

^4^Diagnostic and Interventional Radiology, IRCCS Istituto Ortopedico Rizzoli, Bologna, Italy

^5^Department of Experimental and Clinical Medicine, Section of Anatomy, University of Florence, Italy.

^6^Gut Health Institute Strategic Programme, Quadram Institute Bioscience, Norwich, UK.

^7^School of Health and Medical Sciences, Örebro University, Örebro, Sweden.

^8^Nestlé Research, EPFL Innovation Park, Lausanne, Switzerland.

^9^Institute of Neurological Sciences (IRCCS), Bologna, Italy.

*** Correspondence:**Dr. Serge Rezzi

serge.rezzi@swissvitamin.ch

**** Current address:**

Swiss Vitamin Institute, Épalinges, Switzerland

1. **Supplementary Data**

Amino acid analysis:

The analysis of 34 amino acids in human plasma was performed using an Acquity UPLC I Class system hyphenated to a TQS XEVO triple quadrupole from Waters (Milford, MA, USA). The following amino acids were measured: alanine, β-alanine, α-aminobutyric acid, β-aminoisobutyric acid, γ-aminobutyric acid, arginine, asparagine, aspartic acid, citrulline, assymetric-dimethylarginine, symetric-dimethylarginine, ethanolamine, glutamic acid, glutamine, glycine, histidine, hydroxyproline, isoleucine, leucine, lysine, methionine, 1-methylhistidine, 3-methylhistidine, monomethylarginine, ornithine, phenylalanine, proline, sarcosine, serine, taurine, threonine, tryptophan, tyrosine and valine. The chromatographic system was equipped with a binary pump, a temperature controlled autosampler operated at 20°C and a column oven set to 55°C. Chromatographic separation was achieved by a Waters Acquity AccQ Tag Ultra C18 column (1.7 µm, 100 x 2.1 mm I.D) and an Acquity UPLC BEH C18 VanGuard Pre-column (130Å, 1.7 µm, 2.1 mm x 5 mm) applying a flow rate of 0.7 mL min^-1^. Mobile phase A consisted of 10% AccQ Tag Ultra Eluent A concentrate in H_2_O and AccQ Tag Ultra Eluent B (both from Waters) as mobile phase B. For sample injection, the full loop mode was applied to inject 2 μL of sample. The UPLC system was hyphenated with the mass spectrometer employing electrospray ionization (ESI) in positive mode for ionization. Nitrogen was used as desolvation, nebulization and cone gas while argon was employed as collision gas. Collision energy was adapted to each analyte individually, ranging between 18 and 40 eV performed. Amino acids were identified according to the retention time and compound specific transitions. Quantitation was achieved using an internal standard and an external calibration curve.

Derivatization step: The AccQ•Tag Ultra Reagent (6-aminoquinolyl-N hydroxysuccinimidyl carbamate, or AQC) reacts rapidly with primary and secondary amino acids to form highly stable derivatives that can be stored at room temperature for up to one week. 10 µL of each calibration solution was mixed with 70 µL borate buffer (provided in the AccQ•Tag kit) before 20 µL of the derivatization agent was added. Samples were placed in a heating cabinet for incubation (55°C, 10 minutes) and diluted 20 fold with 0.1 M HCl prior analysis. For preparation of human plasma and QCs, 50 µL of the biological fluid were mixed with ISTD solution and topped up to 200 µl with methanol, 0.1% formic acid (Sigma Aldrich). The mixture was homogenized and centrifuged to remove precipitated proteins. 10 µL of the supernatant was removed, mixed with borate buffer and derivatization agent, incubated at 55°C for 10 min and diluted 20-fold with 0.1 M HCl prior analysis.

Fatty acid analysis:

The quantification of fatty acids in human plasma was performed with the capillary fast gas chromatography (GC) system Agilent 7890A (Agilent Technologies, Santa Clara, CA, USA) equipped with flame ionization detector (FID), a temperature programmable oven applying a temperature gradient ranging from 45°C to 250°C and a split-splitless injector. A capillary column BPX70 from SGE (SGE Europe Ltd, Milton Keynes, UK) with a length of 10 m, an internal diameter of 0.1 mm and a film thickness of 0.2 μm were used for chromatographic separation. Hydrogen 5.7 was applied as carrier gas using a flow rate of 1 mL min^-1^ at 25°C, while hydrogen was used as detector gas. Fatty acids were derivatized by transesterification to form the respective methyl esters (FAME). The transesterification performance (TP) of the method was controlled with TAG 13:0 as second internal standard for plasma samples. FAMEs were identified by retention time and comparison with an external standard. Quantitation was achieved using an internal standard and a response factor (RF) that was evaluated for each fatty acid during method development.

Mineral analysis:

The determination the minerals magnesium (Mg), phosphorous (P), sulfur (S), potassium (K), calcium (Ca), manganese (Mn), iron (Fe), cobalt (Co), copper (Cu), zinc (Zn), selenium (Se), rubidium (Rb), strontium (Sr), molybdenum (Mo) and iodine (I), was carried out using an Agilent 8800 triple quadrupole ICP-MS (Agilent Technologies, Tokyo, Japan) operated in low matrix plasma mode. The mass spectrometric device was equipped with an integrated auto-sampler, a MircoMist nebulizer and a Scott double-pass spray chamber. Elements under evaluation were monitored in three different gas modes (no gas, He or O_2_) to reduce respective spectral interferences for these masses to a minimum. The performance of the mass spectrometer was verified prior analysis by using a multi-element tuning solution. An internal standard (ISTD) solution containing beryllium (200 ng mL^-1^), scandium, gallium, indium, tellurium and bismuth at 100 ng mL^-1^ was added online to the samples using a T-connector. For quantitative analysis, the external calibration approach was applied and a serum certified reference material was analyzed on a daily basis for quality control. Further experimental settings are detailed elsewhere (Konz 2017: J. Proteom. Res.).

# Supplementary Figures and Tables

## Supplementary Figures


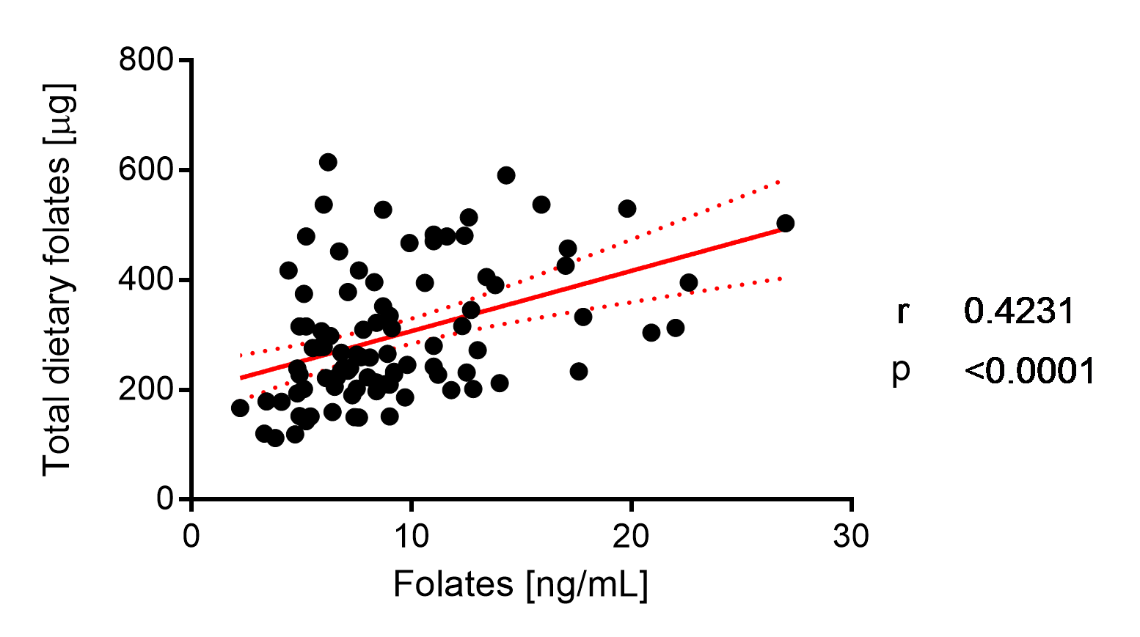


**Supplementary Figure 1: Correlation of dietary folic acid and corresponding blood concentration in females.** Pearson correlation (r) as well as two-tailed p value are shown for females (N=93). Red dotted lines show 95% confidence interval.


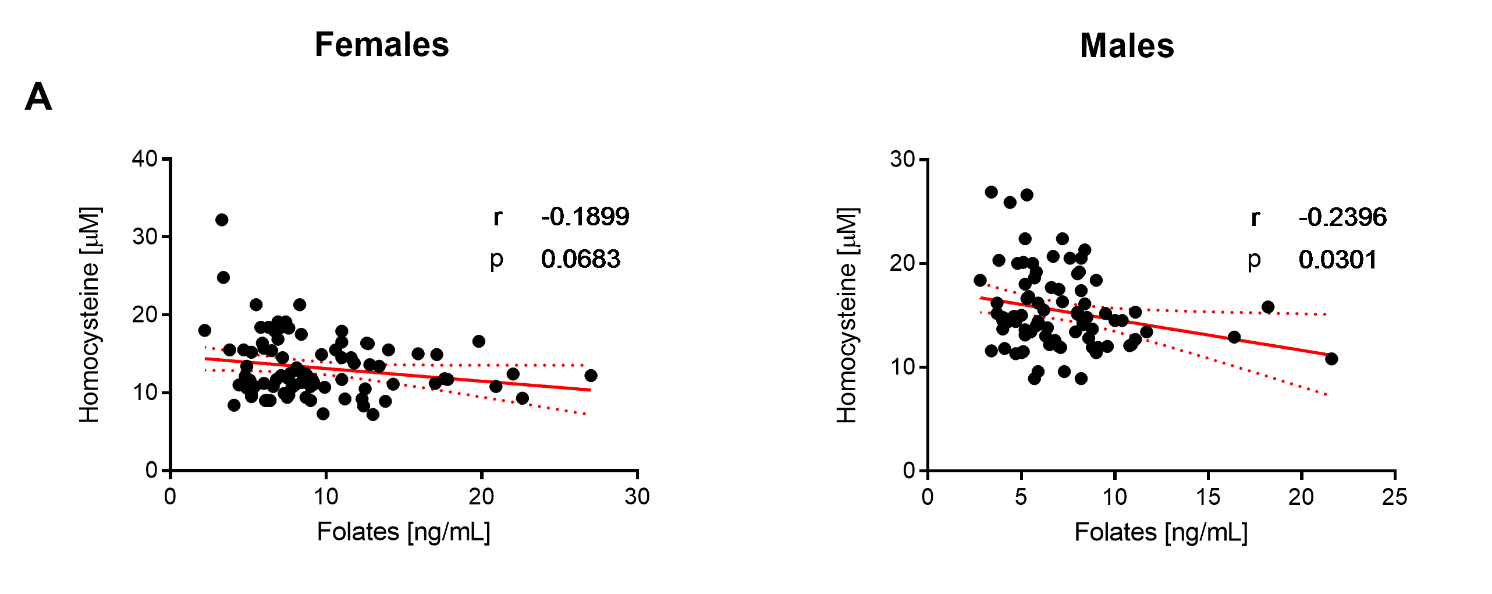


**Supplementary Figure 2: Correlation of homocysteine and folate in the study population.** Pearson correlation (r) as well as two-tailed p value are shown. **(A)** Correlation of homocysteine and folate for females (N=93) and **(B)** Correlation of homocysteine and folate for males (N=83); each dot represents an individual sample. Red dotted lines show 95% confidence interval.

## Supplementary Tables

**Supplementary Table 1)** Comparison of mean daily dietary intake (normalized on body weight) of each food group between men and women.

|  | **Values normalized on body weight** | | | | |
| --- | --- | --- | --- | --- | --- |
|  | **Males**  **(n = 83)** | | **Females**  **(n = 93)** | |  |
| **Food groups** | **Mean^a^** | **S.D.** | **Mean^a^** | **S.D.** | **p^b^** |
| **White grains** | 1.9 | 0.8 | 1.7 | 0.9 | 4.02e-02 |
| **Whole grains** | 0.4 | 0.6 | 0.4 | 0.7 | n.s. |
| **Fruit** | 3.9 | 2.5 | 4.2 | 2.3 | n.s. |
| **Vegetables** | 2.9 | 1.5 | 3.3 | 1.7 | n.s. |
| **Legumes** | 0.2 | 0.3 | 0.2 | 0.3 | n.s. |
| **Dairy products** | 2.4 | 3.2 | 2.5 | 1.5 | n.s. |
| **Cheese** | 0.4 | 0.3 | 0.4 | 0.3 | n.s. |
| **Red and processed meat** | 0.3 | 0.2 | 0.2 | 0.2 | n.s. |
| **White meat** | 0.1 | 0.1 | 0.1 | 0.2 | n.s. |
| **Nuts and seeds** | 0.1 | 0.1 | 0.1 | 0.1 | n.s. |
| **Potatoes** | 0.3 | 0.3 | 0.3 | 0.3 | n.s. |
| **Egg and egg products** | 0.1 | 0.1 | 0.1 | 0.1 | n.s. |
| **Butter and animal fats** | 0.01 | 0.02 | 0.01 | 0.03 | n.s. |
| **Olive oil and other vegetables oils** | 0.2 | 0.1 | 0.2 | 0.1 | n.s. |
| **Sugar-sweetened beverages** | 0.1 | 0.3 | 0.1 | 0.3 | n.s. |
| **Sugar, honey and artificial sweeteners** | 0.1 | 0.1 | 0.1 | 0.1 | n.s. |
| **Sweets, chocolates and snacks** | 0.7 | 0.4 | 0.8 | 0.6 | n.s. |
| **Alcohol (ethanol)** | 0.2 | 0.1 | 0.1 | 0.1 | 1.42e-03 |

^a^Mean daily dietary intake (g day^-1^) normalized on body weight (kg)

^b^Mann-Whitney *U* test; n.s. = not significant
